# Terpenoid indole alkaloid biosynthesis in *Catharanthus roseus*: effects and prospects of environmental factors in metabolic engineering

**DOI:** 10.1007/s10529-021-03179-x

**Published:** 2021-09-25

**Authors:** Yongliang Liu, Barunava Patra, Sanjay Kumar Singh, Priyanka Paul, Yan Zhou, Yongqing Li, Ying Wang, Sitakanta Pattanaik, Ling Yuan

**Affiliations:** 1 Key Laboratory of South China Agricultural Plant Molecular Analysis and Genetic Improvement, Guangdong Provincial Key Laboratory of Applied Botany, South China Botanical Garden, Chinese Academy of Sciences, Guangzhou, 510650 China; 2 University of Chinese Academy of Sciences, Beijing, 100049 China; 3 Department of Plant and Soil Sciences and Kentucky Tobacco Research and Development Center, University of Kentucky, Lexington, Kentucky 40546 USA

**Keywords:** Terpenoid indole alkaloids, *Catharanthus roseus*, Specialized metabolites, Biotic and abiotic factors, Gene regulation, Metabolic engineering

## Abstract

**Abstract:**

Plants synthesize a vast array of specialized metabolites that primarily contribute to their defense and survival under adverse conditions. Many of the specialized metabolites have therapeutic values as drugs. Biosynthesis of specialized metabolites is affected by environmental factors including light, temperature, drought, salinity, and nutrients, as well as pathogens and insects. These environmental factors trigger a myriad of changes in gene expression at the transcriptional and posttranscriptional levels. The dynamic changes in gene expression are mediated by several regulatory proteins that perceive and transduce the signals, leading to up- or down-regulation of the metabolic pathways. Exploring the environmental effects and related signal cascades is a strategy in metabolic engineering to produce valuable specialized metabolites. However, mechanistic studies on environmental factors affecting specialized metabolism are limited. The medicinal plant *Catharanthus roseus* (Madagascar periwinkle) is an important source of bioactive terpenoid indole alkaloids (TIAs), including the anticancer therapeutics vinblastine and vincristine. The emerging picture shows that various environmental factors significantly alter TIA accumulation by affecting the expression of regulatory and enzyme-encoding genes in the pathway. Compared to our understanding of the TIA pathway in response to the phytohormone jasmonate, the impacts of environmental factors on TIA biosynthesis are insufficiently studied and discussed. This review thus focuses on these aspects and discusses possible strategies for metabolic engineering of TIA biosynthesis.

**Purpose of work:**

*Catharanthus roseus* is a rich source of bioactive terpenoid indole alkaloids (TIAs). The objective of this work is to present a comprehensive account of the influence of various biotic and abiotic factors on TIA biosynthesis and to discuss possible strategies to enhance TIA production through metabolic engineering.

## Introduction

**Fig. 1:**
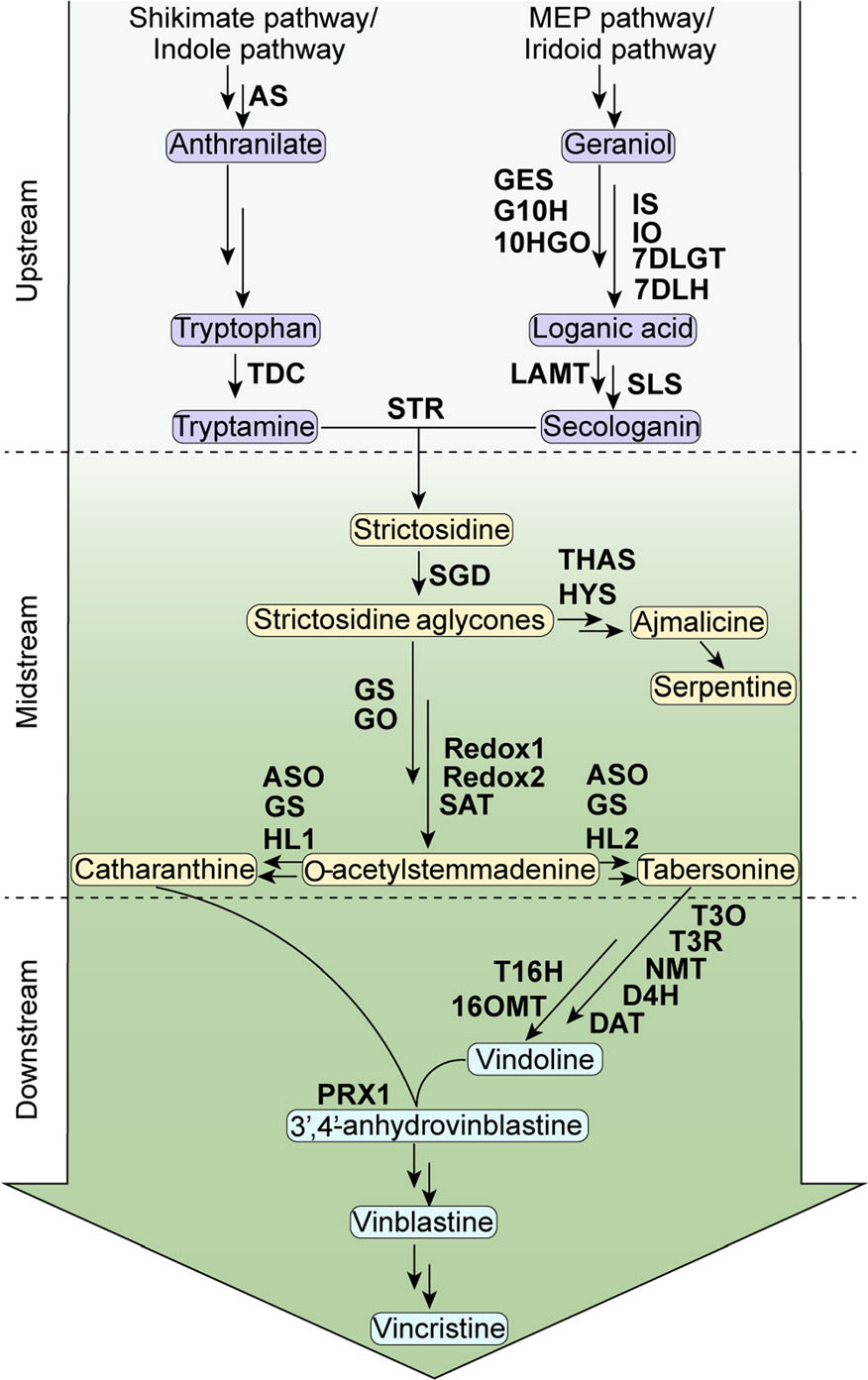
Schematic diagram of the TIA biosynthetic pathway in *C. roseus*, which is divided into three stages: upstream, midstream, and downstream as shown. *ASα* anthranilate synthase, *ASO* *O*-acetylstemmadenine oxidase, *D4H* desacetoxyvindoline-4-hydroxylase, *DAT* deacetylvindoline-4-*O*-acetyltransferase, *7DLGT* 7-deoxyloganetic acid glucosyl transferase, *7DLH* 7-deoxyloganic acid hydroxylase, *G10H* geraniol 10-hydroxylase, *GES* geraniol synthase, *GO* geissoschizine oxidase, *GS* geissoschizine synthase, *HL1/2* hydrolase 1/2, *HYS* heteroyohimbine synthase, *10HGO* 10-hydroxygeraniol oxidoreductase, *IO* iridoid oxidase, *IS* iridoid synthase, *LAMT* loganic acid methyltransferase, *NMT* 3-hydroxy-16-methoxy-2,3-dihydrotabersonine-*N*-methyltransferase, *16OMT* 16-hydroxytabersonine-*O*-methyltransferase, *PRX1* peroxidase 1, *SAT* stemmadenine-*O*-acetyltransferase, *SGD* strictosidine β-glucosidase, *SLS* secologanin synthase, *SS* serpentine synthase, *STR* strictosidine synthase, *T3O* tabersonine 3-oxygenase, *T3R* tabersonine 3-reductase, *T16H2* tabersonine 16-hydroxylase 2, *TDC* tryptophan decarboxylase, *THAS* tetrahydroalstonine synthase

The medicinal plant *Catharanthus roseus* is the source of almost 200 terpenoid indole alkaloids (TIAs), including the anticancer therapeutics vinblastine and vincristine (De Luca et al. 2014). The pharmaceutically important TIAs, vinblastine and vincristine, accumulate in extremely low quantities in *C. roseus*, leading to research efforts to enhance production through various strategies. Towards this end, the TIA biosynthetic pathway has been extensively studied, and the genes encoding key enzymes in the pathway have been identified and characterized (Fig. 1) (Miettinen et al. 2014; Qu et al. 2015, 2018, 2019; Stavrinides et al. 2016). The regulation of the TIA biosynthetic pathway is highly complex and the subject of current research (Patra et al. 2013; Thamm et al. 2016). Biosynthetic genes and transcriptional regulators, either individually or in combination, have been used to engineer the TIA pathway (Sharma et al. 2020; Schweizer et al. 2018; Tang and Pan 2017; Zhao and Verpoorte 2007; Zárate and Verpoorte 2007; Hughes et al. 2004; Hughes and Shanks 2002; Morgan and Shanks 2000; Rijhwani and Shanks 1998; Peebles et al. 2009). As a protocol for regeneration of transgenic *C. roseus* plants is not well established, cell lines and hairy roots are extensively used in the majority of these studies. Recently, transient transformation of *C. roseus* seedlings and flower petals have also been explored (Liu et al. 2019; Schweizer et al. 2018; Singh et al. 2020, 2021). There are only a few reports on the characterization of TIA pathway genes using transgenic plants (Pan et al. 2012; Sharma et al. 2018b). In general, two bioengineering strategies are used to boost TIA production in *C. roseus* (Sharma et al. 2020). One approach is to “push” the metabolic flux towards downstream by increasing the precursor pool through overexpressing genes encoding the upstream or midstream rate-limiting enzymes and associated TFs. The other is to “pull” the metabolic flux towards the final products through manipulating the downstream biosynthetic genes. A more effective approach is perhaps to simultaneously “push-and-pull” by upregulating both upstream and downstream genes. A limitation to such an approach is the requirement of transforming a large number of genes, currently a significant engineering challenge. Increasing evidence shows that certain environmental signals tend to trigger the upstream, midstream, and downstream TIA biosynthetic genes and regulators. Here, we discuss whether the knowledge regarding the impacts of environmental factors on TIA pathway can be explored for metabolic engineering to increase TIA production.

## TIA biosynthetic pathway and the complex gene regulation

The TIA pathway can be broadly divided into three parts: the upstream, midstream, and downstream (Fig. 1). The products of the upstream and midstream pathways, such as strictosidine, ajmalicine, serpentine, catharanthine, and tabersonine, are accumulated in various tissues in the whole plant (van Der Heijden et al. 2004). However, products of the downstream pathway, including vindoline, anhydrovinblastine, vinblastine, and vincristine, are mainly accumulated in the aerial tissues (DeLuca et al. 1986). Two distinct branch pathways provide the precursors for TIA biosynthesis: the shikimate pathway supplies the indole moiety tryptamine, and the methylerythritol pathway (MEP)/iridoid pathway generates the terpenoid moiety secologanin. TIA biosynthesis is highly compartmentalized, occurring in at least four cell types and different subcellular compartments (Courdavault et al. 2014). Biosynthesis of secologanin overlaps between internal phloem associated parenchyma (IPAP) and epidermal cells. Three nitrate/peptide family (NPF) transporters, CrNPF2.4, CrNPF2.5 and CrNPF2.6, are involved in the intracellular transport of multiple iridoid intermediates (Larsen et al. 2017). Biosynthesis of tryptamine occurs in cytosol of the epidermal cells. Secologanin and tryptamine are coupled to form strictosidine in the vacuoles, and then exported to cytosol through the tonoplast-localized NPF transporter CrNPF2.9 (Payne et al. 2017). Strictosidine is then deglucosylated by the nuclear-localized glucosidase, strictosidine ß-D-glucosidase (SGD), to form the strictosidine aglycone, which is converted to reactive dialdehyde that serves as a precursor for the biosynthesis of complex TIAs, including ajmalicine, serpentine, catharanthine, and tabersonine (Guirimand et al. 2010). Catharanthine is secreted out to leaf surface by the ABC transporter CrTPT2 (Yu and De Luca 2013). Tabersonine is further converted to vindoline through a seven-step enzymatic process, occurring in laticifers and idioblasts in the leaf (Qu et al. 2015). Vinblastine and vincristine are derived from the coupling of catharanthine and vindoline. In roots, tabersonine is converted to hörhammericine, catalyzed by tabersonine 6,7-epoxidase isoforms 1 and 2 (TEX1/2), tabersonine 19-hydroxylase (T19H), and tabersonine derivative 19-O-acetyltransferase (TAT) (Carqueijeiro et al. 2018a, b; Giddings et al. 2011).

**Fig. 2:**
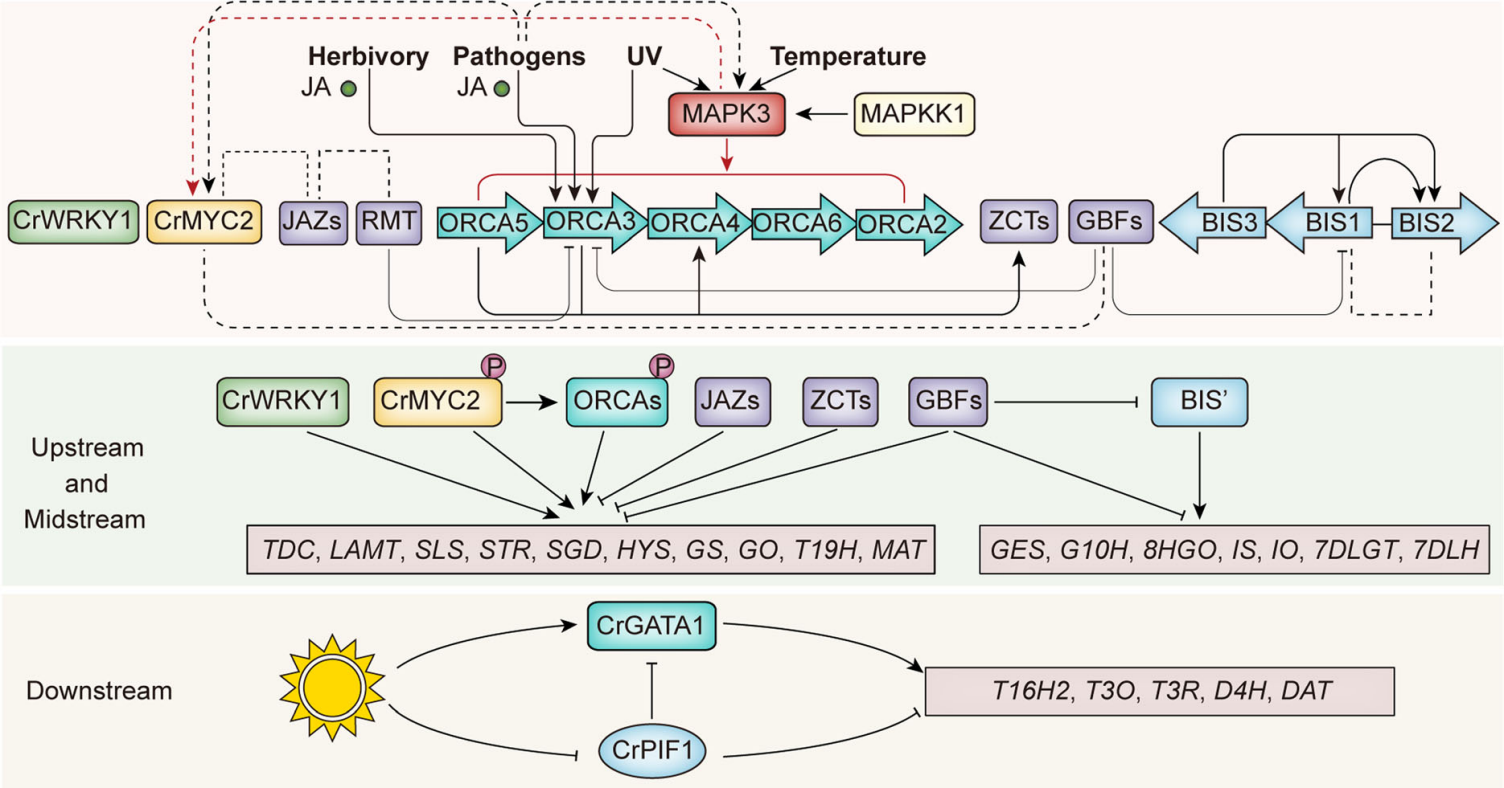
Transcriptional and posttranscriptional regulation of the TIA pathway and influence of environmental factors on TIA biosynthesis. Top panel: complex regulation of the TIA pathways. Environmental factors, such as herbivores, pathogens, UV, and temperature influence TIA biosynthesis by modulating the expression of TFs (e.g., ORCA3 and MYC2) and pathway genes. ORCAs (ORCA2, ORCA, ORCA4, ORCA5, and ORA6) and BIS’ (BIS1, BIS2, and BIS3) are present as clusters in *C. roseus* genome and exhibit intra-cluster regulation (e.g., ORCA5 activates ORCA3 and 4). The ORCAs, CrMYC2, BIS’, CrWRKY1, and CrGATA1 are transcriptional activators, whereas GBFs, ZCTs, JAZs, RMT and CrPIF1 are repressors of the TIA pathway. Middle panel: selective and post-translational regulations of upstream and midstream pathway genes. CrMYC2, ORCAs, and CrWRKY1 regulate upstream indole branch and the midstream pathway genes (from *TDC* to *MAT*), whereas BIS’ regulate the iridoid branch genes (from *GES* to *7DLH*). Many TFs are phosphorylated, leading to functional alteration. The MAPKK1-MPK3 cascade phosphorylate CrMYC2 and ORCAs to regulate their activity. MPK3 expression and kinase activity are induced by pathogens (including fungal endophytes) and temperature. GBFs repress the activities of ORCA3 and BIS1. RMT1 represses the ORCA3 activity on target gene promoters. Lower panel: light regulation of the vindoline pathway. CrGATA1 and CrPIF1 regulate the downstream vindoline pathway. Light (in particular, red light) activates CrGATA1, while causes the degradation of CrPIF1, a repressor of CrGATA1. Protein-protein interaction between CrMYC2-GBFs, CrMYC2-JAZs, RMT-JAZ, and BIS1-BIS2 are indicated by dotted lines. Solid blue arrows indicate direct activation, dotted arrows indicate indirect or unclarified activation, and T-bars represent repression. Phosphorylation of ORCAs and MYC2 is indicated by circled P. *BIS1/2/3* bHLH iridoid synthesis 1/2/3, *MAPKK1* mitogen-activated protein kinase kinase 1, *MPK3* mitogen-activated protein kinase 3, *GBFs* G-box binding factors, *JAZ* jasmonate ZIM domain proteins, *ORCA2/3/4/5/6* octadecanoid-derivative responsive *Catharanthus* AP2-domain, *ZCT *zinc finger *Catharanthus* transcription factors, *CrPIF1* *Catharanthus roseus* phytochrome-interacting factor 1, *RMT1* repressor of MYC2 targets 1, *UV* ultraviolet

The phytohormone jasmonate (JA) and its methyl esters MeJA are key elicitors of TIA biosynthesis. The key components of JA signaling, including the JA co-receptor CORONATINE INSENSITIVE 1 (COI1) and the five JASMONATE ZIM-domain proteins CrJAZ1/2/3/8/10, have been characterized for their roles in regulating TIA biosynthesis (Patra et al. 2018). A number of JA-responsive transcription factors (TFs) have been identified as regulators of the TIA pathway (Fig. 2). These TFs include transcription activators from the TF families of bHLH (CrMYC2, BIS1/2/3) (Zhang et al. 2011; Van Moerkercke et al. 2015, 2016; Singh et al. 2021), AP2/ERF (ORCA2/3/4/5/6, CrERF5) (Singh et al. 2020; Paul et al. 2017, 2020; Pan et al. 2019; van der Fits and Memelink 2000; Li et al. 2013; Menke et al. 1999), and WRKY (CrWRKY1) (Suttipanta et al. 2011), as well as transcription repressors from the TF families of bZIP (GBF1/2) (Sibéril et al. 2001; Sui et al. 2018), zinc finger factors (ZCT1/2/3) (Pauw et al. 2004), bHLH (RMT1) (Patra et al. 2018), and AP2/ERF (CR1) (Liu et al. 2017a). The repressors, ZCTs, RMT1 and GBF1/2, are induced by the transcriptional activators ORCAs, BIS1 and/or CrMYC2 (Sui et al. 2018; Patra et al. 2018; Van Moerkercke et al. 2015; Paul et al. 2017; Peebles et al. 2009). In addition to JA, other phytohormones and environmental factors regulate TIA biosynthesis. Two light-responsive TFs, CrGATA1 and CrPIF1, act as a transcriptional activator and repressor, respectively, to regulate vindoline biosynthesis (Liu et al. 2019) (Fig. 2). However, compared to our understanding of the TIA pathway regulation in response to JA, mechanistic studies on biotic and abiotic factors affecting TIA metabolism are limited. Here, we discuss our current understanding of the effects of environmental factors on TIA biosynthesis.

## Regulation of TIA biosynthesis by environmental factors

### Light

Light regulates plant development and the biosynthesis of many specialized metabolites, such as anthocyanins and artemisinin, mediated by several TFs (Liu et al. 2015; Hao et al. 2019; Li et al. 2016). MAP Kinase 4 (MPK4) and a R2R3 MYB TF, Production of Anthocyanin Pigment 1 (PAP1), regulate light-induced accumulation of anthocyanin in Arabidopsis (Li et al. 2016). Light-induced accumulation of artemisinin in *Artemisia annua* is regulated by the bZIP TF HY5 (Hao et al. 2019). In *C. roseus*, vindoline biosynthesis is regulated by light (Liu et al. 2019; DeLuca et al. 1986). Dark-grown, etiolated *C. roseus* seedlings accumulate a trace amount of vindoline, which increases upon exposure to light (DeLuca et al. 1986). The accumulation of TIAs is correlated with the increase in gene expression and enzyme activities of desacetoxyvindoline-4-hydroxylase (D4H) and deacetylvindoline-4-*O*-acetyltransferase (DAT) upon exposure to light in *C. roseus* seedlings (Table 1) (St-Pierre et al. 1998; De Carolis et al. 1990). The GATA family TF, CrGATA1, is an activator, while CrPIF1 is a negative regulator, of vindoline biosynthesis. Upon exposure of *C. roseus* seedlings to light, CrGATA1 upregulates *tabersonine 16-hydroxylase 2* (*T16H2*), *tabersonine 3-oxygenase* (*T3O*), *tabersonine 3-reductase* (*T3R*), *D4H*, and *DAT*. CrPIF1 represses the expression of *T16H2* and *DAT* in dark. Moreover, CrPIF1 represses the expression of *CrGATA1*. Derepression of CrGATA1, presumably by light-induced degradation of CrPIF1, enhances the expression of five vindoline pathway genes, leading to increased vindoline accumulation (Liu et al. 2019).

### Drought and Salinity

Drought and salt stresses affect plant growth, morphology and metabolic processes. Adaptations to drought and salt stresses involve changes in metabolic processes, including biosynthesis and accumulation of primary and specialized metabolites, that promote drought and salt resistance (Zahedi et al. 2019). In Arabidopsis, drought induces the accumulation of glucosinolates, while salt stress increases the accumulation of flavonoids (Salehin et al. 2019; Li et al. 2019a). In *C. roseus*, drought or salt stress increases the accumulation of TIAs, including ajmalicine, catharanthine (Liu et al. 2017b; Jaleel et al. 2008a, b, c), vindoline, vinblastine, and vincristine (Liu et al. 2017b; Amirjani 2013; Osman et al. 2007; Fatima et al. 2015; Dutta et al. 2013; Ababaf et al. 2021) (Table 1). Consistent with the increase of TIAs, expression of both upstream (*TDC* and *STR*) and downstream (*D4H* and *DAT*) TIA pathway genes are induced by drought or salt stress. However, it is unclear how these pathway genes are regulated by stress signal transduction and gene transcription.

Phytohormones play important roles in abiotic stress response in plants (Ullah et al. 2018). Abscisic acid (ABA) is the key phytohormone which intensifies drought and salt tolerance in plants. The SnRK2 protein kinases and protein phosphatases 2 C (PP2C) are important components of the ABA signaling pathway. Under normal conditions (low ABA content), PP2Cs interact with and dephosphorylate SnRK2s to inhibit ABA response. When the ABA level increases in response to drought or salt stress, PP2C dissociate from SnRK2 which is auto-phosphorylated and then phosphorylate the downstream targets to promote ABA responses (Ullah et al. 2018). In response to ABA, a SnRK2 kinase from *A. annua* (AaAPK1) phosphorylates a bZIP TF, AabZIP1, to activate artemisinin biosynthesis, while a PP2C-type phosphatase, AaPP2C1, negatively regulates artemisinin biosynthesis through dephosphorylation of AaAPK1 (Zhang et al. 2018, 2019). ABA also promotes catharanthine production in *C. roseus* suspension cells (Chen et al. 2013). It is possible that drought or salt stress triggers ABA signaling that activates SnRK2s to promote TIA accumulation.

**Table 1:** The effects of environmental factors on TIA biosynthesis in *C. roseus*

Environmental factors	Plant materials	Regulated genes	Metabolites	References
Light	Seedlings	DAT (↑)*	Vindoline (↑)*	DeLuca et al. (1986), St-Pierre et al. (1998)
	Seedlings	D4H (↑)	–	De Carolis et al. (1990)
	Seedlings	CrPIF1 (↑), CrGATA1 (↑), T16H2 (↑), T3O (↑), T3R (↑), D4H (↑), DAT (↑)	Vindoline (↑)	Liu et al. (2019)
Drought	Shoots	–	Vincristine (↑)	Osman et al. (2007)
	Roots	–	Ajmalicine (↑)	Jaleel et al. (2008b)
	Seedlings	–	Total TIAs (↑)Vinblastine (↑)Vincristine (↑)	Amirjani (2013)
	Leaves	STR (↑)	–	Dutta et al. (2013)
	Seedlings	TDC (↑), STR (↑), DAT (↑)	Catharanthine (↑)Vindoline (↑)Vinblastine (↑)	Liu et al. (2017b)
	Leaves	–	Vinblastine (↑)Vincristine (↑)	Ababaf et al. (2021)
Salt	Shoots	–	Vincristine (↑)	Osman et al. (2007)
	Roots	–	Ajmalicine (↑)	Jaleel et al. (2008c)
	Roots	–	Ajmalicine (↑)	Jaleel et al. (2008a)
	Leaves	–	Total TIAs (↓)Vinblastine (↓)Vincristine (↓)	Idrees et al. (2011)
	Leaves	STR (↑)	Catharanthine (↓)Vindoline (↓)Vinblastine (↓)Vincristine (↑)	Dutta et al. (2013)
	Leaves	D4H (↑), DAT (↑)	–	Mokhaberi et al. (2013)
	Cultivated tissues	–	Vinblastine (↑)Vincristine (↑)	Fatima et al. (2015)
Hight temperature	Leaves	–	Catharanthine (↑)Vindoline (↑)Vinblastine (↑)	Guo et al. (2007)
	Leaves	CrMPK3 (↑)	–	Raina et al. (2013)
Low temperature	Leaves	TDC (↓), D4H (↓)	Catharanthine (↓)Vindoline (↓)Vinblastine (↓)	Dutta et al. (2007)
	Leaves	STR (↓)	Catharanthine (↓)Vindoline (↓)Vinblastine (↓)Vincristine (↓)	Dutta et al. (2013)
Ultraviolet	Leaves	TDC (↑), STR (↑)	Total TIAs (↑)	Ouwerkerk et al. (1999a, b)
	Suspension cells	TDC (↑), STR (↑)	Catharanthine (↑)	Ramani and Chelliah 2007)
	Suspension cells	–	Catharanthine (↑)Vindoline (↑)	Ramani and Jayabaskaran 2008)
	Hairy roots	G10H (↑)	Total TIAs (↑)	Binder et al. (2009)
	Seedlings	–	Catharanthine (↑)Vindoline (↑)Vinblastine (↑)	Guo et al. (2014)
	Leaves	G10H (↑), TDC (↑), STR (↑), ORCA3 (↑), T16H (↑), D4H (↑), DAT (↑)	Strictosidine (↑)Ajmalicine (↑)Catharanthine (↑)Vindoline (↑)	Zhu et al. (2015)
	Cultivated plantlets	–	Vincristine (↑)	Salama et al. (2020)
	Leaves	–	Ajmalicine (↑)Vinblastine (↑)Vincristine (↑)	Zhong et al. (2021)
Heavy metal Vanadium	Suspension cells	–	Ajmalicine (↑)Catharanthine (↑)	Smith et al. (1987)
Cadmium	Leaves	–	Catharanthine (↑)Vindoline (↑)Vinblastine (↑)	Chen et al. (2018)
	Leaves and roots	–	Ajmalicine (↓)Vindoline (↓)	Srivastava and Srivastava (2010)
	Suspension cells	TDC (↑)	Ajmalicine (↑)	Zheng and Wu (2004)
Nickel, manganese	Roots and leaves	–	Ajmalicine (↓)Vindoline (↓)	Srivastava and Srivastava (2010)
Lead	Leaves	–	Vindoline (↓)	Srivastava and Srivastava (2010)
Chromium	Shoots	–	Vinblastine (↓)Vincristine (↑)	Rai et al. (2014)
Cobalt	Suspension cells	–	Total TIAs (↑)	Fouad et al. (2018)
Nutrient deficiency Nitrogen, phosphorus, magnesium, sulfur	Roots	–	Ajmalicine (↓)	Mendonça Freitas et al. (2016)
Potassium	Roots	–	Ajmalicine (↑)	
Herbivore(*Manduca sexta*)	Leaves	ORCA3 (↑), STR (↑), SGD (↑), D4H (↑), DAT (↑)	Total TIAs (↑)Ajmalicine (↑)Catharanthine (↑)Vindoline (↑)	De Bernonville et al. (2017)
Pathogens *Aspergillus niger*, *Fusarium moniliforme*, *Trichoderma viride*	Suspension cells	–	Ajmalicine (↑)	Namdeo et al. (2002)
*Fusarium oxysporum*	Suspension cells	TDC (↑)	Total TIAs (↑)	Tang et al. (2011)
*Aspergillus flavus*	Callus tissues	–	Vinblastine (↑)Vincristine (↑)	Tonk et al. (2016)
Yeast extract	Callus tissues	–	Vinblastine (↑)Vincristine (↑)	Maqsood and Abdul 2017)
*Pseudomonas fluorescens*, *Azospirillum brasilense*	Roots	TDC (↑), STR (↑)	–	Ahmadzadeh et al. (2020)
*Curvularia *sp. CATDLF5, *Choanephora infundibulifera* CATDLF6	Leaves	G10H (↑), TDC (↑), STR (↑), 16OMT (↑), D4H (↑), DAT (↑), PRX1 (↑), ORCA3 (↑), ZCTs (↓)	Vindoline (↑)	Pandey et al. (2016)

Up and down arrows indicate increase and decrease of gene expression and metabolite accumulation, respectively

### Temperature

Both low and high temperature limit plant growth and development by reprograming various metabolic processes. Temperature affects the accumulation of specialized metabolites, such as flavonoids and phenolic compounds, which possibly play roles in temperature tolerance (Cohen and Kennedy 2010; Chalker-Scott 1999). In Arabidopsis, anthocyanin accumulation is induced by low temperature and suppressed by high temperature (Kim et al. 2017). Artemisinin biosynthesis in *A. annua* is also induced by cold and regulated by a TF module comprising the TFs, AabHLH112 and AaERF1 (Xiang et al. 2019). In *C. roseus* leaves, accumulation of midstream and downstream metabolites, including catharanthine, vindoline and vinblastine, is increased by high temperature (Guo et al. 2007) and suppressed by low temperature (Dutta et al. 2007, 2013) (Table 1). Consistent with the cold-induced suppression of metabolites, expression of *STR*, *TDC*, and *D4H* is also decreased (Dutta et al. 2007, 2013). Interestingly, a heat-activated MAPK, CrMAPK3 (Raina et al. 2013), induces the expression both upstream (*TDC* and *STR*) and downstream (*D4H* and *DAT*) TIA biosynthetic genes in *C. roseus* leaves (Raina et al. 2012) (Fig. 2). We have reported that CrMAPK3 and CrMAPK6 likely phosphorylate CrMYC2 and ORCAs to induce TIA biosynthetic genes, such as *TDC* and *STR* (Paul et al. 2017). In Arabidopsis, MAPK3 and MAPK6 are important components in cold signaling pathway (Li et al. 2017). These findings suggest that severe temperature possibly regulate TIA biosynthesis through the CrMAPK3/6 signaling pathway.

### Ultraviolet

Ultraviolet (UV) radiation (200–400 nm) can be classified into UV-A (320–400 nm), UV-B (280–320 nm), and UV-C (200–280 nm). Accumulation of specialized metabolites, such as phenolic compounds and flavonoids, serves as a common protective mechanism against potentially damaging UV irradiation to plants (Zhang and Björn 2009; Frohnmeyer and Staiger 2003). Exposure of Arabidopsis seedlings to UV-B (8.0 kJ m^− 2^ day^− 1^) for 6 h significantly induces the expression of key phenylpropanoid pathway genes, such as phenylalanine ammonia-lyase (PAL) and chalcone synthase (CHS). Longer exposure to UV increases the accumulation of flavonoids and sinapate compounds, suggesting their roles in UV protection (Li et al. 1993). Short-term (14 days) exposure to UV-B (4.2 kJ m^− 2^ day^− 1^) and UV-C (5.7 kJ m^− 2^ day^− 1^) also induces accumulation of flavonoids and artemisinin in leaves and inflorescences of *A. annua* (Rai et al. 2011). In the medicinal plant water mint (*Mentha aquatica*), prolonged UV-B exposure (2 or 4 h daily for 3 weeks) alters the volatile oil profile and increases the accumulation of phytochemicals (Nazari and Zarinkamar 2020). TIAs are known to absorb UV and function as UV protectants (Ouwerkerk et al. 1999b). UV-B induces the accumulation of ajmalicine, catharanthine, and vindoline in *C. roseus* suspension cells or hairy roots (Table 1) (Binder et al. 2009; Ramani and Jayabaskaran 2008; Ramani and Chelliah 2007). UV-A or UV-B irradiation also leads to increased accumulation of strictosidine, catharanthine, vindoline, vinblastine, and vincristine in *C. roseus* leaves or shoot cultures (Salama et al. 2020; Guo et al. 2014; Ouwerkerk et al. 1999b; Zhu et al. 2015). UV treatment induces the expression of pathways genes, including *G10H*, *10HG*O, *TDC, STR*, *T16H*, *D4H*, and *DAT*, and the TF *ORCA3* (Ouwerkerk et al. 1999a; Binder et al. 2009; Zhu et al. 2015) (Fig. 2). A recent study on the effects of UV-B on the mitochondria and plastid proteomes of *C. roseus* shows the increase of proteins related to the MEP pathway, that provides the monoterpene precursor. Additionally, consistent with the previous reports, UV-B exposure increases accumulation of ajmalicine, vincamine, deacetylvindoline, and vincristine in *C. roseus* leaves (Zhong et al. 2021). These findings collectively suggest that the UV-B receptor and the associated signal transduction pathway are involved in the regulation of the TIA pathway. Another study shows that expression and kinase activity of CrMAPK3 are induced by UV-C irradiation in *C. roseus* leaves (Raina et al. 2012), indicating that UV-induced TIA biosynthesis is possibly regulated by protein kinases (Fig. 2). Supporting this hypothesis, a recent study shows that UV-B exposure increases ATP content in *C. roseus* leaves and induces significant change in leaf phospho-proteome. Upon UV exposure, phosphoproteins related to protein synthesis/degradation/ modification, heat-shock proteins, and protein kinases, such as the calcium-dependent protein kinases, change significantly (Zhong et al. 2019).

### Heavy metals

Studies show that heavy metals affect TIA accumulation in *C. roseus*. In suspension cells, vanadium (V), cadmium (Cd), and cobalt (Co) induce the production of ajmalicine and catharanthine (Table 1) (Zheng and Wu 2004; Smith et al. 1987; Fouad and Hafez 2018). Expression of *TDC* is induced by Cd in *C. roseus* suspension cells, which correlates to the increase of TIAs (Zheng and Wu 2004). Cd is also reported to induce the accumulation of catharanthine, vindoline and vinblastine in *C. roseus* leaves (Chen et al. 2018); however, this is in contrary to a previous study showing Cd, reduces vindoline contents in *C. roseus* leaves (Srivastava and Srivastava 2010). Chromium (Cr) treatment leads to an increase of vinblastine and vincristine in *C. roseus* leaves (Rai et al. 2014). Ni and Mn reduce vindoline content in leaves. Cadmium (Cd), Nickel (Ni) or manganese (Mn) treatment increases serpentine content by 2–3 fold, while suppresses ajmalicine in *C. roseus* roots (Srivastava and Srivastava 2010).

### Nutrient deficiency

Nutrient deficiency affects not only plant growth and development, but also the biosynthesis of many specialized metabolites (Yang et al. 2018). For example, nutrient deficiency results in increased accumulation of anthocyanins in plants (Zhang et al. 2017; Wang et al. 2015; Ren et al. 2021). Nitrogen (N) is an essential nutrient for plant growth and development and a constituent of alkaloids. N fertilizers affect the accumulation of TIAs in *C. roseus* plants (Table 1) (Gholamhosseinpour et al. 2011). N deficiency reduces ajmalicine accumulation in *C. roseus* roots (Mendonça Freitas et al. 2016), while higher N supply reduces contents of catharanthine, vindoline and vinblastine in *C. roseus* leaves (Guo et al. 2014). In addition to N, deficiency of other nutrients also alters TIA production. Potassium (K) deficiency increases, while deficiencies of phosphorus (P), magnesium (Mg), and sulfur (S) decrease, ajmalicine accumulation in *C. roseus* roots (Mendonça Freitas et al. 2016).

### Herbivores and pathogens

The plant specialized metabolites are defense molecules that confer resistance against pathogens and herbivores (Panda et al. 2021). Similarly, *C. roseus* produces TIAs in response to herbivore and pathogens as chemical defense (Fig. 2). TIAs, such as catharanthine and anhydrovinblastine, are toxic to herbivores and pathogens (De Bernonville et al. 2017; Roepke et al. 2010). The TIA pathway metabolites and corresponding genes are induced by the herbivory of *Manduca sexta* on *C. roseus* leaves (Table 1) (De Bernonville et al. 2017). In *C. roseus* suspension cells, the fungal pathogens, *Aspergillus niger*, *Fusarium moniliforme*, *F. oxysporum* and *Trichoderma viride*, induce TDC activity and the accumulation of total alkaloids (Tang et al. 2011; Namdeo et al. 2002). In *C. roseus* calli, yeast extract or *A. flavus* induces the accumulation of vinblastine and vincristine (Maqsood and Abdul 2017; Tonk et al. 2016). In *C. roseus* leaves, the fungal endophytes *Curvularia sp*. CATDLF5 and *Choanephora infundibulifera* CATDLF6 upregulate the expression of TIA pathway genes and the accumulation of vindoline (Pandey et al. 2016). In addition, expression of *TDC* and *STR* is induced in *C. roseus* roots after infection by two rhizobacteria, *Pseudomonas fluorescens* and *Azospirillum brasilense* (Ahmadzadeh et al. 2020).

The phytohormones JA, salicylic acid (SA), and ethylene are involved in plant disease resistance (Dong 1998). These phytohormones crosstalk with the MAPK cascades to confer disease resistance (Wang et al. 2013; Han et al. 2010; Zhang and Liu 2001). The homologous MAPK3 and 6 are emerging as key components in disease resistance by regulating various defense responses, including the induction of camalexin, a phytoalexin in Arabidopsis (Meng and Zhang 2013; Mao et al. 2011). We have also reported the critical roles of the CrMAPKK1-CrMAPK3/6 cascade in the regulation of TIA biosynthesis in *C. roseus* (Paul et al. 2017). Furthermore, in addition to the well characterized JA induction, the disease resistance associated hormones, SA and ethylene, also induce the production of TIAs in *C. roseus* leaves or seedlings (Soltani et al. 2020; Wang et al. 2016; Pan et al. 2010, 2015; Idrees et al. 2011; El-Sayed and Verpoorte 2004). Meanwhile, both upstream (*G10H*, *TDC* and *STR*) and downstream (*T16H*, *D4H* and *DAT*) genes are upregulated by SA and ethylene. Therefore, biotic factors possibly trigger TIA accumulation through the sophisticated crosstalk between phytohormone signaling and the CrMAPKK1-CrMAPK3/6 pathway.

## Strategies for metabolic engineering TIA biosynthesis

The low-level accumulation of therapeutically important TIAs has intrigued researchers to develop innovative strategies to boost TIA production. Previous studies on the TIA pathway have identified the genes encoding enzymes and regulatory TFs. The genes encoding enzymes and TFs have been used for metabolic engineering of the TIA pathway with various degrees of success. Studies on the influences of environmental factors have provided limited but important information on changes in the expression profiles of TIA pathway genes and regulators. Here, we discuss whether the biotic or abiotic factor-responsive pathway genes and/or TFs can be used as tools to engineer TIA biosynthesis. In this section, we also describe several technology platforms and strategies used for TIA pathway engineering.

### Resources

Both homologous and heterologous gene expression systems have been used to study the regulation and metabolic engineering of TIA pathway. As the generation of stable transgenic *C. roseus* plants is not well established, suspension cells and hairy roots serve as effective platforms for studying TIA biosynthesis and regulation. However, the cell lines or hairy roots only produce upstream and midstream metabolites due to the lack of precursors or extremely low expression of the upstream pathway genes, limiting their uses in engineering of downstream TIAs. For instance, some of the cell lines (e.g., MP183L) do not produce any alkaloid under normal cultural conditions. *ORCA3* overexpression induces tryptamine, but artificial feeding of the cell lines with the terpenoid precursor loganin is necessary to produce the downstream TIAs. Additionally, biosynthesis of vindoline and dimeric alkaloids vincristine and vinblastine do not occur in the cell lines (van der Fits and Memelink 2000; Zhang et al. 2011). However, it has been demonstrated that cambial meristematic cell cultures of *C. roseus* can overcome some obstacles of traditional suspension cells, allowing the accumulation of the downstream TIAs, including vindoline, vinblastine, and vincristine (Moon et al. 2015, 2018), making meristematic cell culture a promising platform for TIA engineering. In addition, young *C. roseus* seedlings (Weaver et al. 2014; Mortensen et al. 2019), leaves (Raina et al. 2012; Sharma et al. 2018a), and flower petals (Schweizer et al. 2018) have also been used for transient overexpression of genes to reprogram both upstream and downstream metabolism.

Heterologous systems, such as yeast and tobacco plants, have also been successfully used to produce several therapeutic metabolites, such as artemisinin from *Artemisia annua* (Farhi et al. 2011), taxadine from *Taxus spp*. (Li et al. 2019b), noscapine from *Papaver somniferum* (Li et al. 2018; Li and Smolke 2016), cannabinoids from *Cannabis sativa* (Luo et al. 2019), and certain intermediates of TIAs (Miettinen et al. 2014; Qu et al. 2015). The iridoid and indole branch of the TIA pathway has been reconstructed in *Nicotiana benthamiana* to produce strictosidine (Miettinen et al. 2014), whereas the yeast cells expressing seven-step vindoline pathway are able to produce vindorosine and vindoline (Qu et al. 2015). However, the heterologous systems come with various limitations. The TIA pathway requires more than 30 enzymes in different cellular compartments. Engineering the whole pathway in a heterologous system is therefore cumbersome. The other drawback is the limitation or absence of precursors, which requires introduction of additional genes or precursor feeding to overcome. For instance, production of strictosidine in *N. benthamiana* leaves requires two additional enzymes, geranyl diphosphate synthase and geraniol synthase, to boost the precursors as well as supplementation of iridoid intermediates (Miettinen et al. 2014). Similarly, tobacco cell suspension culture overexpressing *TDC* and *STR* produces strictosidine only after feeding with secologanin (Hallard et al. 1997). Vindoline and vindorosine are produced in yeast cells expressing the seven vindoline pathway genes upon feeding with tabersonine (Qu et al. 2015). Moreover, some downstream TIAs are highly cytotoxic, *C. roseus* has evolved spatial separation of specific intermediates and transporters for intracellular transport and secretion. We thus argue that a homologous system, such as meristematic cells, young seedlings, hairy roots, or transgenic *C. roseus* plants, is more suitable for TIA bioengineering.

### Technologies and Tools

Gene overexpression and RNAi-mediated silencing are widely used for studying metabolic pathways in plants, including *C. roseus* (Zhao and Verpoorte 2007; Jaggi et al. 2011; Paul et al. 2017, 2020; Liu et al. 2019; Patra et al. 2018; Suttipanta et al. 2011). Virus-induced gene silencing (VIGS) has emerged as an effective tool to study the regulation of TIA biosynthesis in *C. roseus* leaves and flowers (Liscombe and O’Connor 2011; Sung et al. 2014; Liu et al. 2019; Patra et al. 2018, 2021). Recently, an improved *C. roseus* VIGS method has been developed, in which the target gene and the visual marker gene have been incorporated in the same plasmid to successfully identify the silenced tissues *in planta* (Yamamoto et al. 2021). Furthermore, the generation of stable transgenic *C. roseus* plants have also been reported (Sharma et al. 2018b; Pan et al. 2012; Wang et al. 2012). The reproducible generation of stable transgenic plants will enable the *in planta* bioengineering by targeting upstream, midstream, and downstream pathway genes using overexpression, RNAi, and genome-editing (e.g., using CRISPR-Cas9).

TFs are attractive engineering tools as they regulate a subset or all genes in a metabolic pathway. TFs alone, or in combination with key enzymes, have been used to engineer TIA pathway with various degrees of success (Sharma et al. 2020; Pan et al. 2012; Wang et al. 2010). Compared to using individual TFs, combined expression of three TFs *ORCA3*, *BIS1*, and a mutant *MYC2* significantly upregulates TIA pathway gene expression and increases TIA accumulation in *C. roseus* flower petals (Schweizer et al. 2018). However, combined overexpression of the three TFs has no effect on downstream TIAs such as vindoline. Although studies on the influence of environmental factors on TIA biosynthesis are limited to a few pathway genes and regulators, they provide important information on the changes in gene expression profiles and TIA accumulation. Additionally, several biotic and abiotic factors have broad effects to TIA biosynthesis, not only to up- and mid-stream metabolites, but also to downstream TIAs, e.g., vindoline. In the following section, we discuss several strategies used previously for engineering TIA biosynthesis. We also describe how the environmental factor-responsive genes can be used as tools to boost TIA production using similar strategies.

### Engineering to increase the upstream TIA precursors

Genes encoding several rate-limiting enzymes and TFs, either alone or in combination, have been used to increase the accumulation of upstream metabolites in *C. roseus* hairy roots or suspension cells. Overexpression of *STR* in suspension cells greatly induced the accumulation of ajmalicine, serpentine, catharanthine, and tabersonine (Canel et al. 1998). Similarly, combined overexpression of *TDC* and an Arabidopsis *anthanilate synthase* (*ASα*) in hairy roots enhanced the production of tryptamine and serpentine (Hughes et al. 2004). Co-expression of *G10H* and *ORCA3*, either in hairy roots or transgenic plants, improved the production of TIAs (Wang et al. 2010; Pan et al. 2012). In addition to using pathway enzyme genes, overexpression of TFs, such as *ORCA4* or *ORCA5*, in hairy roots significantly induced the accumulation of ajmalicine, catharanthine, and tabersonine (Paul et al. 2017, 2020). Although the effects of environmental factors on regulatory genes have not been well studied, the expression of many key upstream pathway genes, such as *TDC*, *STR*, and *G10H*, are altered by environmental factors, such as drought, salt, low temperature, and UV, leading to change in TIA accumulation. These findings suggest that increasing upstream precursors using the key pathway genes or the TFs regulating them will lead to increase of TIA accumulation.

### Pushing the metabolic flux towards downstream

Manipulation of the upstream pathway genes can push the metabolite flux to downstream. For instance, transient overexpression of *TDC* and *STR* in *C. roseus* leaves induced expression of downstream pathway genes, including *DAT* and *PRX1*, and increases the production of vindoline and vinblastine (Sharma et al. 2018a). Co-expression of *ORCA3* and *G10H* in *C. roseus* plants not only increased the accumulation of the midstream metabolites ajmalicine and catharanthine, but also the downstream vindoline (Pan et al. 2012). Expression of *TDC*, *STR*, *D4H*, and *DAT* was altered by various external factors, such as UV and herbivory, leading to the increase in midstream and downstream TIAs, suggesting their potentials for increasing TIA production.

### Pulling the metabolic flux to downstream

Metabolic flux can be pulled towards downstream by manipulating the downstream TIA biosynthetic steps. Overexpression of the key vindoline pathway gene *DAT* in *C. roseus* plants increased the production of vindoline (Wang et al. 2012). Transient overexpression of the transcription activator *CrGATA1* in seedlings improved vindoline accumulation (Liu et al. 2019). Knocking down the expression of the transcription repressor *CrPIF1* in leaves by VIGS also improved vindoline accumulation (Liu et al. 2019). Expression of *CrGATA1* and other vindoline pathway genes is affected by light. The light-induced vindoline and the dimeric TIAs, such as vinblastine and vincristine, are accumulated in aerial parts of the plants. The genes encoding downstream enzymes, such as *D4H* and *DAT*, or TFs, such as *CrGATA1*, may be co-overexpressed with *PRX1* either in seedlings or transgenic plants to boost TIA production. Alternatively, the meristematic cell culture, that is capable of producing the dimeric TIAs, can be used to test this strategy.

### Increasing the downstream TIAs through a push-and-pull strategy

The production of downstream TIAs can be maximized through combination of push and pull strategies. Overexpression of the transcriptional activator *CrERF5* in *C. roseus* petals induces the expression of the upstream *TDC* and *STR*, as well as the downstream *D4H* and *PRX1* (Pan et al. 2019). VIGS of the transcription repressor *CR1* in *C. roseus* leaves also upregulates *TDC, STR, DAT*, and *PRX1* (Liu et al. 2017a). Similarly, transient overexpression of the kinase *CrMAPK3* in *C. roseus* leaves upregulates the expression of *TDC*, *STR, D4H*, and *DAT* (Raina et al. 2012). However, it is unclear whether CrERF5 or CR1 directly regulates both upstream and downstream genes, but rather, regulates only one subset of the pathway genes such that the following metabolite flux affects the other subset of the genes. Additionally, whether the expression of these known regulatory genes is affected by environmental factors requires further study. Therefore, detailed analysis of spatio-temporal expression profiles of known regulators in response to different environmental stimuli will provide additional tools for TIA metabolic engineering. Expression of many upstream and downstream TIA pathway genes, such as *STR*, *TDC*, *G10H*, *DAT* and *D4H*, is altered by UV, salt, high temperature, and herbivory, leading to the increase in dimeric alkaloid and its precursors such as vindoline and catharanthine. Therefore, combined overexpression of upstream and downstream pathway genes responsive to environmental factors will potentially boost TIA production.

Regulatory factors associated with UV-B signal transduction are well characterized in Arabidopsis (Morales et al. 2013; Rizzini et al. 2011). In Arabidopsis, the UV-B receptor UVR8 regulates expression of the genes involved in UV protection and defense response, as well as biosynthesis and signaling of JA and SA. The UV receptor and other regulatory factors in the UV signaling pathway are conserved across plant species (Tossi et al. 2019), and UV induces the accumulation of both upstream and downstream TIAs. Therefore, the signaling components associated with the UV-B pathway can be potential targets to increase TIAs in *C. roseus*.

## Conclusions

Biosynthesis of many specialized metabolites is affected by environmental factors (Li et al. 2020; Yang et al. 2018). One notable example is anthocyanins often found in fruits, vegetable and flowers (Maier et al. 2013; Plunkett et al. 2019; Xie et al. 2012). The accumulation of other specialized metabolites, such as artemisinin, is also affected by low light, temperature, and UV (Xiang et al. 2019; Hao et al. 2019; Pan et al. 2014). Systematic studies on the influence of environmental factors led to the identification key regulatory genes and the underlying molecular mechanisms governing biosynthesis of these metabolites. The anticancer drugs vinblastine and vincristine are in demand but produced in extremely low quantities in *C. roseus* leaves. Attempts to increase TIAs through metabolic engineering met with various degrees of success. Studies on environmental factors clearly show that drought, salt, light, and temperature affect the production of both upstream and downstream TIAs in *C. roseus*. The increase or decrease of TIA accumulation in response to environmental factors is likely a consequence of the changes in the expression of pathway genes, regulators, and signal transduction components, such as protein kinases. Gene regulation of TIA biosynthesis is highly complex. However, a comprehensive mechanistic study on how environmental factors regulate pathway gene expression to affect TIA biosynthesis is lacking. In the past few years, a number of genes encoding key pathway enzymes, kinases, and regulators in the TIA pathway have been identified and characterized. Transporters play key roles in the intracellular transport of TIA intermediates. However, the influence of environmental factors on TIA transporters and the newly identified genes have not been studied. Moreover, many repressors involved in the regulation of the TIA pathway have been discovered recently (Shoji and Yuan 2021; Patra et al. 2018; Pauw et al. 2004; Sui et al. 2018). The repressors, working in concert with the activators, enable *C. roseus* to dial the amplitude of TIA biosynthesis. Expression profiles of these repressors in response to environmental factors will provide important insights on TIA regulation. The past engineering approaches heavily rely on overexpression of positive regulators and key enzymes. Overexpression of a positive regulator while knockdown or knockout of a repressor could be an alternative strategy to engineer TIA biosynthesis. RNA-sequencing has emerged as a powerful tool to study transcriptomic landscape in response to any biotic or abiotic factors. Transcriptomic analyses in response light, JA, and UV provided important information on factors involved in artemisinin biosynthesis (Hao et al. 2017; Pan et al. 2014). *C. roseus* transcriptomic analyses also led to the identification of new pathway genes and regulators. The majority of published studies focus on individual environmental factor on TIA accumulation. However, plants are subject to many biotic and abiotic stress factors in a natural environment. “Stress combination transcriptomics” attempt to dissect the plant responses to different combinations of biotic and/or abiotic stresses (Zandalinas et al. 2020). Study on combined effects of environmental factors on specialized metabolism is still lacking. Generation and analyses of transcriptomes of *C. roseus* in response to different environmental factors will allow further elucidation of the regulation of TIA pathway, thus generating potential candidates for metabolic engineering.
